# Cardiac and Proprioceptive Accuracy Are Not Related to Body Awareness, Perceived Body Competence, and Affect

**DOI:** 10.3389/fpsyg.2020.575574

**Published:** 2021-01-14

**Authors:** Áron Horváth, Luca Vig, Eszter Ferentzi, Ferenc Köteles

**Affiliations:** ^1^Doctoral School of Psychology, ELTE Eötvös Loránd University, Budapest, Hungary; ^2^Institute of Health Promotion and Sport Sciences, ELTE Eötvös Loránd University, Budapest, Hungary

**Keywords:** proprioception, cardioception, interoceptive accuracy, interoceptive sensibility, affect, body awareness

## Abstract

Interoception in the broader sense refers to the perception of internal states, including the perception of the actual state of the internal organs (visceroception) and the motor system (proprioception). Dimensions of interoception include (1) interoceptive accuracy, i.e., the ability to sense internal changes assessed with behavioral tests, (2) confidence rating with respect to perceived performance in an actual behavioral test, and (3) interoceptive sensibility, i.e., the self-reported generalized ability to perceive body changes. The relationship between dimension of cardioceptive and proprioceptive modalities and their association with affect are scarcely studied. In the present study, undergraduate students (*N* = 105, 53 males, age: 21.0 ± 1.87 years) filled out questionnaires assessing positive and negative affect (Positive and Negative Affect Schedule), interoceptive sensibility (Body Awareness Questionnaire), and body competence (Body Competence Scale of the Body Consciousness Questionnaire). Following this, they completed a behavioral task assessing cardioceptive accuracy (the mental heartbeat tracking task by Schandry) and two tasks assessing proprioceptive accuracy with respect to the tension of arm flexor muscles (weight discrimination task) and the angular position of the elbow joint (joint position reproduction task). Confidence ratings were measured with visual analog scales after the tasks. With the exception of a weak association between cardioceptive accuracy and the respective confidence rating, no associations between and within modalities were found with respect to various dimensions of interoception. Further, the interoceptive dimensions were not associated with state and trait positive and negative affect and perceived body competence. In summary, interoceptive accuracy scores do not substantially contribute to conscious representations of cardioceptive and proprioceptive ability. Within our data, non-pathological affective states (PANAS) are not associated with the major dimensions of interoception for the cardiac and proprioceptive modalities.

## Introduction

Interoception refers to the processing of information originating from within the body (Cameron, 2002). Originally, it was a synonym for visceroception; later, the inclusion of somatosensory and proprioceptive information was also proposed (Vaitl, 1996; Ceunen et al., 2016; Berntson et al., 2018). The current paper applies this broad approach to interoception. Thus, conscious aspects of interoception include body sensations associated with emotions, awareness of non-emotive body processes and the perception of the actual state of the locomotor system.

The recently accepted conceptualization of conscious aspects of interoception describes at least two major dimensions (Ceunen et al., 2013; Garfinkel and Critchley, 2013; Garfinkel et al., 2015a). Interoceptive accuracy (IAc or sensitivity) refers to the acuity of perception of internal changes and states as assessed by behavioral methods. Its self-report counterpart, i.e., the perceived performance in an a behavioral test of acuity, is called confidence. Finally, the perceived general ability to sense body changes is called interoceptive sensibility (IS) or awareness in the literature. Empirical evidence shows that the association between these three dimensions of cardiac interoception is weak or non-existing (see below).

It is worth noting that there is an inconsistency in the literature with respect to the concept of interoceptive sensibility. Unfortunately, it is not clear which questionnaires should be used to assess the dispositional aspect of interoceptive sensibility. Garfinkel et al. (2015a) recommend the Body Awareness Scale of the Body Perception Questionnaire (Porges, 1993). The Body Awareness Questionnaire (BAQ) (Shields et al., 1989) and the Multidimensional Assessment of Interoceptive Awareness (Mehling et al., 2012) have been also used in the literature (Meessen et al., 2016; Ferentzi et al., 2019). Although the former does not make a distinction between visceroception and proprioception, whereas the latter includes only visceroceptive modalities, a recent study indicated a substantial overlap between the two constructs (Ferentzi et al., 2020).

Concerning the emotional experience, the primary importance of visceroception has been suggested by many authors (James, 1884, James, 1890; Lange, 1885; Damasio, 1994), whereas others emphasize the role of the somatosensory system (Darwin, 1872; Tomkins, 1962, 1981; Izard, 1971). These models assume the causal role of interoceptive information in the development of affective experience thus they are called peripheral theories of emotion. Central theories do not suppose such a causal link (Cannon, 1927, 1931; Panksepp, 1982, 1991; Oatley and Johnson-laird, 1987; Davis, 1989; LeDoux, 1990); still, they accept that emotions are typically characterized by peripheral changes that prepare the organism for the behavioral response. As a proportion of these changes, both visceral and somatosensory, may reach conscious awareness, an association between the emotional experience and the perception of body changes can be explained by central theories too.

Cardiac response plays a central role in the physiological component of emotional reactions, as they are usually characterized by an increased energetic demand (Lacey and Lacey, 1978). It is widely assumed that, in line with the tenets of peripheral theories of emotion, the accuracy of perception of cardiac activity, dubbed cardioceptive accuracy, contributes to the emotional experience (Wiens et al., 2000; Pollatos et al., 2005). On the other hand, a more intense emotional reaction (e.g., if it is accompanied by sympathetic activation) can improve the perception of heartbeats (Schandry et al., 1993; O’Brien et al., 1998; Fairclough and Goodwin, 2007). Empirical studies revealed a positive association between the intensity (arousal) component of emotions and cardioceptive accuracy (Wiens et al., 2000; Barrett et al., 2004; Pollatos et al., 2005, 2007b; Herbert et al., 2007, 2010). Also, improved cardiac accuracy was found to be related to the actual level of anxiety in a number of studies (Schandry, 1981; Ludwick-Rosenthal and Neufeld, 1985), whereas no such associations were reported in others (Pollatos et al., 2007a; Werner et al., 2013). From a theoretical point of view cardioceptive confidence may also contribute to the affective experience. For example, manipulated feedback on heart rate was enough to intensify the emotional reaction (Valins, 1966, 1967). Even more intriguingly, if actual and perceived heart rate did not correspond, the latter influenced the perceived level of arousal (Thornton and Hagan, 1976; Kerber and Coles, 1978; Woll and McFall, 1979; Parkinson, 1985).

Proprioceptive information is also assumed to play a substantial role in the formation of emotional experience. According to different theories (for a review, see Moors, 2009), somatic and/or motor changes, modulated by cognitive processing, are cornerstones of the arising of affective feelings. Changes in the musculoskeletal system can modulate the emotional experience. Shafir et al. (2015) showed that different affective feelings can be evoked by specific, complex movement patterns. Power posing may also change the affective experience; however results with respect to behavioral (more risk-taking) and hormonal responses (i.e., increased testosterone and decreased cortisol level) are controversial (Carney et al., 2010; Ranehill et al., 2015; Simmons and Simonsohn, 2017). In the same vein, EMG activity increases in many muscles and muscle groups in stressful situations (Lundberg et al., 1994; Wahlström et al., 2002; Krantz et al., 2004; Luijcks et al., 2014), and it is possible to reduce stress and anxiety through relaxation techniques (e.g., progressive relaxation, autogenic training), which operate (at least partially) through the systematic relaxation of muscles (Kanji et al., 2006; Rausch et al., 2006). Finally, Cacioppo et al. (1993) showed that the activation of arm flexor muscles activates the approach system, which biases the judgment of neutral stimuli to the positive direction. By contrast, activation of arm extensors stimulates the avoidance system, resulting in the opposite effect. Neumann and Strack (2000) drew a similar conclusion in a categorization task. Overall, these findings support the idea that the actual state of muscles can impact the emotional experience.

Based on the aforementioned role of proprioceptive information in the formation of the affective experience, it is logical to assume that, similar to cardioceptive information, individual differences in the accuracy of processing of proprioceptive information (aka proprioceptive accuracy) are related to differences in emotional processing. In accordance with this assumption, alterations in processing and integration of proprioceptive input can be associated with pathological conditions. For example, a greater reliance on proprioception during the completion of a motor task is associated with impairments in imitation and empathy in autism spectrum, and attention deficit hyperactivity disorder (Gao et al., 2019). In fibromyalgia, however, patients were found to be less reliant on proprioceptive information than healthy controls (Bardal et al., 2016). Also, decreased proprioceptive accuracy was found in chronic pain (Tsay et al., 2015) and schizophrenia (Rosenbaum et al., 1959, 1965; Leventhal et al., 1982; Chang and Lenzenweger, 2005). In contrast, somatoform disorders are accompanied by higher proprioceptive accuracy (Scholz et al., 2001). It is also important to note that an emotionally intense state, e.g., the high level of stress, decreases proprioceptive accuracy (Şenol et al., 2018). However, not all studies confirmed the aforementioned relationships, there are null findings too, for example with respect to schizophrenia (Ritzler and Rosenbaum, 1974; Ritzler, 1977), fibromyalgia (Akyol et al., 2013; Ulus et al., 2013), and chronic pain (Tsay et al., 2015). Moreover, Horváth et al. (2019) found that there is no association between trait affect and proprioceptive accuracy, as assessed with the Joint Position Reproduction test in the elbow joint. Additionally, proprioceptive accuracy was not correlated with body awareness–a construct that overlaps with interoceptive sensibility (Ferentzi et al., 2020)–and perceived body competence (Ferentzi et al., 2017; Horváth et al., 2019).

When investigating the role of interoceptive accuracy, it is a fundamental question whether individual characteristics in information processing established in one modality (e.g., cardioception) can be generalized to other modalities (e.g., proprioception). Ferentzi et al. (2018) reported no association between modalities of interoception. A significant association was found only between measures within the same modality for three visceroceptive modalities (i.e., pain threshold and tolerance, gastric fullness and unpleasantness, and the intensity and unpleasantness of bitter taste), but there was no association between the two included measures of proprioceptive accuracy (ipsilateral and contralateral version of the joint position reproduction test in the elbow joint). These and other results (Garfinkel et al., 2017) show that interoceptive accuracy cannot be generalized across interoceptive modalities.

With respect to joint-related proprioceptive accuracy, a number of measurement paradigms were developed (Han et al., 2016). Studies investigating the association between different tests in one joint (Barrack et al., 1984; Grob et al., 2002; Jong et al., 2005; Elangovan et al., 2014; Li et al., 2016; Niespodziński et al., 2018; Yang et al., 2020) consistently report that accuracy is test-specific. The same conclusion can be drawn with respect to cardioception: accuracy scores obtained by heartbeat discrimination methods that use forced-choice methods and methods that use heartbeat tracking (i.e., counting) typically show no or only weak associations (Pennebaker and Hoover, 1984; Weisz et al., 1988; Phillips et al., 1999; Schaefer et al., 2012; Hart et al., 2013; Schulz et al., 2013; Michal et al., 2014; Garfinkel et al., 2015a,b; Forkmann et al., 2016; Ring and Brener, 2018). Moreover, proprioceptive measurement methods can be conducted with respect to different joints; Han et al. (2013) and Waddington and Adams (1999) revealed that accuracy, assessed with the same paradigm (active movement extent discrimination apparatus) is joint-specific, and only the same joints of the left and right side of the body show an association. The actual exertion (or tension) of muscles represents another proprioceptive modality; a fundamental difference is that activation of the muscles is controlled by a feed-forward mechanism thus the efferent information plays a similarly important role in the processing of the actual state as the afferent input (Miall and Wolpert, 1996; Cullen, 2004). Further, joint-related acuity primarily relies on receptors located in the joints (Ruffini end organs), whereas muscle-related accuracy is impacted by afference from receptors in the muscles (muscle spindles) (Batson, 2009; Jha et al., 2017).

Confidence rating, the self-reported dimension of interoception, appears to be independent of interoceptive accuracy for healthy participants (e.g., Ehlers et al., 1995). In another study, accuracy and confidence were associated among high performers in both applied heartbeat perception tasks, i.e., the mental tracking and the discrimination task (Garfinkel et al., 2015a). The authors interpret their results as a dissociation of the assessed dimensions of interoception which was replicated by others regarding the mental tracking task (Forkmann et al., 2016; Meessen et al., 2016). A weak positive association between cardioceptive accuracy and confidence was found in another study (Köteles et al., 2020a). Besides heartbeat perception, interoceptive confidence with respect to respiration has been also investigated and the dissociation between accuracy and confidence was confirmed (Garfinkel et al., 2016). In the field of proprioception, however, confidence has not been assessed to date.

The major goal of the present study is to shed more light on the associations within and between the dimensions of cardioception and two modalities of proprioception, i.e., the sense of joint position and muscle tension. We also wanted to explore the associations between affect and the behavioral and self-report measures of these modalities.

The following hypotheses were tested. First, accuracy and confidence show a weak positive association within the same modality (H1). Second, accuracy and confidence between modalities are independent of each other (H2). Third, cardioceptive accuracy and confidence are associated with affect, whereas proprioceptive accuracy and confidence are not (H3). Finally, we assumed that proprioceptive accuracy and confidence would not be associated with perceived body competence and body awareness (H4).

## Materials and Methods

### Participants

*A priori* sample size calculation for *r* = 0.3, α = 0.05 (one-tailed), 1-β = 0.9 indicated a minimum required sample size of *N* = 92 (Faul et al., 2007). Participants were undergraduate students of Eötvös Loránd University (*N* = 105, 53 males, age: 21.0 ± 1.87 years, 95 right handed). Participants consuming alcohol and/or taking psychoactive drugs within 8 h before the experiment, and those with severe injury/disability of the arm were excluded. Participation was rewarded with partial course credit. Joint Position Reproduction test was missing for nine individuals due to technical problems. The study was approved by the Research Ethics Committee of the university. Before participation, everyone signed an informed consent form.

### Behavioral Measures

#### Proprioceptive Accuracy–Joint Position Sense

Joint Positions Sense was assessed with a version of the Joint Position Reproduction Test (JPR) (Goble, 2010), where participants had to reproduce elbow joint positions. We tested the non-dominant arm of the participants. Participants were blindfolded, seated, and asked to keep a standard posture (upper arms parallel with the ground and in line with the body). During the measurement, they placed their upper arm on a rotatable lever, which was connected to a motor, and made possible the accurate (±0.1 degree) measurement and movement of the elbow joint. They had to hold a handle and keep their hand on a button. 180 degree indicated fully extend elbow. From starting position, the machine moved the arm of the participant to the target positions with a speed of 12 degree/s. After spending 4 s there, the device moved back the lever to the starting position. After 1 s, the lever started to move again, with a speed of 8 degree/s. The task of the participants was to press the button, when they felt that their arm reached the target position. Following this, the lever moved the arm back to the starting position again and a new trial begun. The starting position was always 160 degree, while the target position changed from trial to trial. Overall, nine trials were conducted, with nine different target positions (150, 135, 120, 105, 90, 75, 60, 45, and 30 degree). Every target position was presented once; the order of presentation was randomized. To calculate the accuracy of Joint Position Reproduction, an error score (i.e., the difference between the target and the reproduced position (i.e., the difference between the target and the reproduced position) was calculated for each trial. Outliers above and below two standard deviation were removed and missing values were imputed by using the fully conditional specification (MCMC) and linear regression model options of SPSS v20 software. To determine accuracy, we used two error scores: constant and variable error (Schutz and Roy, 1973; Boisgontier et al., 2012; Goble et al., 2012). Constant error is the mean of the error scores and shows the magnitude and direction of the systematic distortion in position judgments. Negative values of constant error score indicate bias toward the inside direction, whereas positive values indicate bias toward the outside direction. Internal consistency of constant error was acceptable (Cronbach’s alpha: 0.751). Variable error is the standard deviation of the error scores and shows the inconsistency of judgments (i.e., higher variable error shows higher level of inconsistency.

#### Proprioceptive Accuracy–Weight Discrimination

To assess weight discrimination ability, participants had to compare the weight of two objects (Chang and Lenzenweger, 2005). These objects were glass bottles filled with water, identical in shape and size. During the measurement, participants eyes’ were covered; they had to keep a standing posture, keep their left upper arm next to their body, and their lower arm in a flexion of approximately 90 degree.

Overall, 32 comparisons were made. The weight of one of the presented bottles was always 200 g. In one half of the trials (16), the other bottle was 200 g (identical pairs), while in the other half (16), it was 215 g (different pairs). The presentation order of the pairs and that of the bottles within pairs were randomized. Participants had to hold every bottle for 8 s, verbally judge if they were the same weight or one was heavier. For heavier judgments, it also had to be indicated which weight was heavier. Weight discrimination ability was calculated by dividing the number of correct trials by the number of all trials.

#### Cardioceptive Accuracy

Cardioceptive accuracy was assessed with a mental heartbeat-counting paradigm (Schandry, 1981). Participants had to count their heartbeats silently, while sitting on a chair, with their hands on their laps. They were explicitly encouraged to count if they had the lightest heartbeat sensation in any part of their body but were also asked not to count if they did not have any sensation. After a practice trial, which lasted for 15 s, three test trials of different lengths (25, 35, and 50 s) were conducted. The test trials were presented in a randomized order. The number of heartbeats were recorded with the NeXus recording system (NeXus Wireless Physiological Monitoring and Feedback: NeXus-10 Mark II, Version 1.02; BioTrace + Software for NeXus-10 Version: V201581; Mind Media BV, Herten, the Netherlands). For every interval, an accuracy score was calculated as: 1–| (HB recorded–HB counted)/HB recorded|. For every individual, the scores of the three intervals were averaged to calculate cardioceptive accuracy. Internal consistency of the Schandry task was high (Cronbach’s alpha = 0.906).

### Questionnaires and Questions

#### Confidence Ratings

After every task (Joint Position Sense, Weight Discrimination, Cardioceptive accuracy), participants’ subjective judgment about their performance (“*How do you think you performed in this test?*”) was recorded. For this purpose, they had to indicate their perceived performance on a 10 cm-long, vertical visual analog scale. The anchor points were “*The best possible*” and “*The worst possible*.” We measured the distance of the crossed part of the line from the bottom of the visual analog scale in millimeters. Higher values indicate higher levels of confidence.

#### Interoceptive Sensibility–Body Awareness

Body Awareness Questionnaire measures the self-reported sensitivity to bodily processes, and the ability to anticipate bodily reactions (Shields et al., 1989; Köteles, 2014). Participants have to answer 18 questions on seven-point Likert-scales (e.g., “I notice distinct body reactions when I am fatigued”), where higher scores mean higher levels of body awareness (except one reversed item). Internal reliability in this sample was good (Cronbach’s α = 0.848).

#### Body Competence

Body Competence was assessed with the Body Competence Scale of Miller’s Private and Public Body Consciousness Questionnaire (Miller et al., 1981). The scale consists of four questions (e.g., “*I’m better coordinated than most people*”), rated on a five-point Likert scale. Higher values indicate higher levels of perceived physical competence. Internal consistency of the scale in this study was good (Cronbach’s α = 0.835).

#### Affect

We used the Positive and Negative Affect Schedule (PANAS) to asses affect (Watson, 1988; Gyollai et al., 2011). The questionnaire can be used with two different instructions, to measure state and trait aspects of affect. The questionnaire is divided into two subscales, positive affect (PA) (e.g., enthusiasm), and negative affect (NA) (e.g., nervousness); both measured with 10 items. Participants have to rate how intensely they feel the given emotional state on a five-point Likert scale, from 1 (“*Very slightly or not at all*”) to 5 (“*Very much*”). Higher scores refer to higher levels of positive and negative affect, respectively. Cronbach’s α values indicated acceptable to high levels of internal consistency in this study (Positive Trait: 0.872, Positive State: 0.922, Negative Trait: 0.854, Negative State: 0.794).

### Procedure and Statistical Analysis

Data was collected in two phases. Participants had to fill out the questionnaires (with the exception of state PANAS) at home in an online form. The order of the questionnaires was: demographic data, trait PANAS, BAQ, Body Competence. Behavioral measures were conducted individually in the laboratory in a randomized order. Before the behavioral tasks, participants had to fill out the state PANAS questionnaire. Statistical analysis was conducted using the Jasp v0.11 software (JASP Team, 2019) using both the frequentist and Bayesian approach. Due to violations of the requirement of normality, associations were estimated using non-parametric correlations, i.e., Spearman’s rho in the frequentist analysis and Kendall’s Tau in the Bayesian analysis. For the Bayesian analysis, values below 0.33 indicated the superiority of the null-hypothesis, and values over 3 indicated the superiority of the alternative hypothesis (Wetzels and Wagenmakers, 2012).

## Results

Descriptive statistics of the assessed variables are presented in Table 1.

**TABLE 1:** Descriptive statistics of the assessed variables.

***N* = 105**	**M ± SD**	**Min–max**
Cardioceptive accuracy	0.51 ± 0.270	0.0 to 0.963
Cardioceptive confidence	46.80 ± 26.042	1 to 96
Weight discrimination accuracy	15.66 ± 3.622	9 to 29
Weight discrimination confidence	40.74 ± 22.419	0 to 94
Joint Position Sense–constant error	6.5 ± 4.652	−11.341 to 21.386
Joint Position Sense–variable error	6.76 ± 2.277	2.287 to 12.352
Joint Position Sense confidence	61.094 ± 22.239	0 to 98
State NA	12.54 ± 3.190 (15.8 ± −5.9)*	10 to 24
Trait NA	17.22 ± 5.181 (19.5 ± 6.0)*	10 to 36
State PA	31.60 ± 8.247 (29.0 ± 8.0)*	12 to 49
Trait PA	36.429 ± 6.090 (35.7 ± 6.2)*	17 to 50
Interoceptive sensibility–BAQ	80.95 ± 15.449	40 to 122
Body competence	14.45 ± 3.581	4 to 20

Weight discrimination and proprioception of the angle of the elbow joint were conducted with the subdominant hand. NA, negative affect; PA, positive affect; BAQ, Body Awareness Questionnaire. *Normative data reported by Watson and Clark (1994).

**FIGURE 1:**
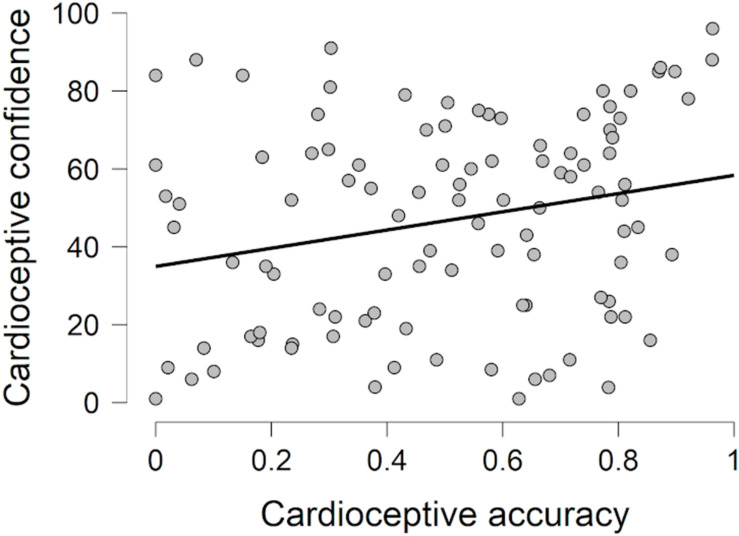
Association between cardioceptive accuracy and cardioceptive confidence ratings.

No significant associations but one between accuracy and confidence ratings (H1) within the included interoceptive modalities were revealed (Table 2). For cardioception, accuracy was weakly (*r*_*s*_ = 0.25, *p* < 0.05) related to confidence (Figure 1); this was supported by the Bayesian analysis (BF_10_ = 5.063). The null model (i.e., the lack of association) was more probable for all other modalities.

**TABLE 2:** Correlations between accuracy and confidence for the three interoceptive modalities.

**Interoceptive modality**	**Spearman correlation (p)**	**Bayesian Kendall’s Tau (BF_10_)**
Cardioception	0.25* (0.011)	0.182 (5.063)
Weight discrimination	0.05 (0.643)	0.032 (0.144)
Joint Position Sense–constant error	−0.09 (0.433)	−0.058 (0.191)
Joint Position Sense–variable error	−0.05 (0.640)	−0.050 (0.176)

*p < 0.05.

Concerning associations between indicators of accuracy (H2), no significant correlation was found. Only the two joint position sense related indices (i.e., constant and variable error) showed a moderate association (*r*_*s*_ = 0.41, *p* < 0.001; *r*_τ_ = 0.28, BF_10_ = 500.816); Bayesian analysis indicated the superiority of the null hypothesis for all other cases (Table 3).

**TABLE 3:** Correlations between accuracies for the three interoceptive modalities.

***N* = 105**	**Cardioception**	**Weight discrimination**	**Joint Position Sense–constant error**	**Joint Position Sense–variable error**
Cardioception	–	0.00 (0.987)	−0.08 (0.455)	−0.09 (0.399)
Weight discrimination	−0.003 (0.127)^+^	–	0.09 (0.395)	−0.01 (0.938)
Joint Position Sense–constant error	−0.058 (0.188)^+^	0.059 (0.188)^+^	–	0.41 (<0.001)***
Joint Position Sense–variable error	−0.051 (0.174)^+^	−0.004 (0.133)^+^	0.281 (500.816)***	–

Upper triangle: frequentist analysis with Spearman’s rho coefficients (*p*-values); Lower triangle: Bayesian analysis with Kendall’s Tau coefficients (BF^10^ values). ***p < 0.001/BF10 > 100. +: BF10 < 0.33.

With respect to confidence ratings, no significant association was found between cardioceptive and weight discrimination related confidence (*r*_*s*_ = 0.09, *p* = 0.374; *r*_τ_ = 0.067, BF_10_ = 0.212), between cardioceptive and joint position sense related confidence (*r*_*s*_ = 0.14, *p* = 0.198; *r*_τ_ = 0.092, BF_10_ = 0.314), and joint position sense and weight discrimination related confidence (*r*_*s*_ = −0.07, *p* = 0.518; *r*_τ_ = −0.037, BF_10_ = 0.155). Again, Bayesian analysis indicated the superiority of the null hypothesis for all cases (Table 4).

**TABLE 4:** Correlations between confidence ratings for the three interoceptive modalities.

***N* = 105**	**Cardioception**	**Weight discrimination**	**Joint Position Sense**
Cardioception		0.09 (0.374)	0.135 (0.198)
Weight discrimination	0.07 (0.212)^+^		−0.07 (0.518)
Joint Position Sense	0.09 (0.314)^+^	−0.04 (0.155)^+^	

Upper triangle: frequentist analysis with Spearman’s rho coefficients (*p*-values); Lower triangle: Bayesian analysis with Kendall’s Tau coefficients (BF^10^ values). ***: *p* < 0.001/BF10 > 100. +: BF10 < 0.33.

Between measures of interoception and questionnaire scores (H3, H4), correlation analysis indicated no significant correlations (Table 5). Bayesian analysis indicated the superiority of the null hypothesis for most of the cases and was inconclusive (i.e., in the 0.33–3 domain) for the remaining associations (Table 6).

**TABLE 5:** Associations between indicators of interoceptive accuracy and confidence ratings and questionnaire scores.

***N* = 105**	**State NA**	**Trait NA**	**State PA**	**Trait PA**	**BAQ**	**Body competence**
Cardioceptive accuracy	0.128 (0.192)	0.065 (0.512)	−0.041 (0.674)	−0.007 (0.947)	0.020 (0.840)	−0.10 (0.331)
Cardioceptive confidence	−0.026 (0.797)	−0.065 (0.519)	0.024 (0.809)	0.132 (0.187)	0.112 (0.262)	0.0 (1)
Weight discrimination–accuracy	0.044 (0.658)	−0.099 (0.316)	−0.021 (0.834)	−0.114 (0.248)	0.043 (0.664)	−0.019 (0.850)
Weight discrimination–confidence	−0.163 (−0.163)	−0.131 (0.189)	0.131 (0.189)	0.041 (0.681)	0.171 (0.084)	0.012 (0.900)
Joint Position Sense–constant error	−0.064 (0.534)	−0.026 (0.802)	−0.152 (0.140)	−0.123 (0.231)	0.117 (0.255)	0.005 (0.963)
Joint Position Sense–variable error	−0.078 (0.448)	0.005 (0.640)	−0.103 (0.317)	−0.075 (0.465)	−0.161 (0.177)	−0.138 (0.179)
Joint Position Sense–confidence	0.048 (0.646)	0.019 (0.854)	0.085 (0.414)	0.027 (0.794)	0.185 (0.073)	0.070 (0.503)

*Frequentist analysis, Spearman’s rho coefficients (*p* values). None of the associations reached the *p* < 0.05 level of significance. NA, negative affect; PA, positive affect; BAQ, Body Awareness Questionnaire.*

**TABLE 6:** Associations between indicators of interoceptive accuracy and confidence ratings and questionnaire scores.

***N* = 105**	**State NA**	**Trait NA**	**State PA**	**Trait PA**	**BAQ**	**Body competence**
Cardioceptive accuracy	0.102 (0.412)	0.044 (0.159)^+^	−0.026 (0.318)^+^	−0.006 (0.128)^+^	0.011 (0.129)^+^	−0.07 (0.223)^+^
Cardioceptive confidence	−0.021 (0.136)^+^	−0.045 (0.161)^+^	0.020 (0.135)^+^	0.095 (0.346)	0.079 (0.258)^+^	0.001 (0.129)^+^
Weight discrimination accuracy	0.030 (0.141)^+^	−0.067 (0.211)^+^	−0.014 (0.130)^+^	−0.081 (0.269)^+^	0.033 (0.144)^+^	−0.019 (0.133)^+^
Weight discrimination–confidence	−0.123 (0.680)	−0.088 (0.305)^+^	0.093 (0.337)	0.024 (0.137)^+^	0.107 (0.462)	0.012 (0.131)^+^
Joint Position Sense–variable error	−0.06 (0.195)1^+^	4.507e-4 (0.133)^+^	−0.063 (0.201)^+^	−0.052 (0.176)^+^	−0.106 (0.422)	−0.095 (0.341)
Joint Position Sense–constant error	−0.051 (0.174)^+^	−0.018 (0.137)^+^	−0.107 (0.462)	−0.081 (0.264)^+^	0.085 (0.278)^+^	0.003 (0.133)^+^
Joint Position Sense–confidence	0.034 (0.150)^+^	0.014 (0.137)^+^	0.060 (0.193)^+^	0.014 (0.137)^+^	0.126 (0.677)	0.048 (0.169)^+^

*Bayesian analysis, Kendall’s Tau coefficients (BF^10^ values). +: BF10 < 0.33. NA, negative affect; PA, positive affect; BAQ, Body Awareness Questionnaire.*

## Discussion

The goal of the present study was to investigate the associations between different modalities (cardioception and two proprioceptive modalities) of interoception and their dimensions (accuracy, confidence ratings and sensibility). Associations with positive and negative affect and perceived body competence were also investigated. Overall, accuracy and confidence were associated with respect to the cardiac modality only; further, no between-modality associations and associations with interoceptive sensibility, affect, and body competence were found.

### Interoceptive Accuracy and Confidence

Contrary to our first hypothesis, accuracy and confidence were found to be independent of each other with respect to the two proprioceptive modalities. In other words, people are not able to sense their actual performance in these tasks. In the cardioceptive modality, however, similar to previous studies (Garfinkel et al., 2015a), we found a weak positive association between accuracy and confidence. This latter finding is in line with the insight that top-down information substantially impacts performance in the mental tracking task (Ring et al., 2015; Ring and Brener, 2018; Zamariola et al., 2018; Desmedt et al., 2020). Although a strict instruction was applied (i.e., participants were explicitly encouraged not to count if they did not have any sensation to report), which presumably decreases the impact of top-down factors (Ehlers et al., 1995; Desmedt et al., 2018), the involvement of conscious processes in the tracking task is substantial. If one combines knowledge on the usual frequency of his or her heartbeats with the number and timing of actually sensed and counted heartbeats, performance in the task can be estimated (Desmedt et al., 2020). In the case of the proprioceptive modalities, however, no such information is available, thus actual and perceived accuracy show complete dissociation.

In line with our second hypothesis, interoceptive accuracy and the respective confidence ratings proved to be modality-specific. We replicated the findings of Ferentzi et al. (2018), namely that cardioceptive accuracy, as assessed with the mental heartbeat tracking task, does not correlate with measures of proprioceptive accuracy (joint position reproduction and weight discrimination tests in this study). Moreover, in accordance with the findings of other studies (Barrack et al., 1984; Grob et al., 2002; Jong et al., 2005; Elangovan et al., 2014; Li et al., 2016; Niespodziński et al., 2018; Yang et al., 2020), no association between accuracies with respect to two proprioceptive modalities was found. This lack of association might reflect the actual independence of the two abilities; however, conceptual differences (i.e., the weight discrimination test does not involve a reproduction element and it was measured with a forced choice paradigm) can also explain this finding.

The confidence-related findings were similar to those for accuracy: there were no associations between cardioceptive and proprioceptive tasks, and between the two proprioceptive tasks. Empirical results concerning interoceptive confidence across modalities are scarce. Garfinkel et al. (2015a) found a strong positive association between confidence ratings of two heartbeat perception tasks (i.e., mental heartbeat tracking task and the discrimination task). In our data, this indicates that the perception of performance is not only more or less independent of actual performance, but also differs between modalities; cardioception-related confidence rating show closer connection than those of proprioceptive confidence. This also suggests that top-down factors that usually impact perception, such as previous experiences and expectations, may show considerable modality-specific differences.

### Interoception and Affect

Contrary to our expectation (H3), we did not find any association between state and trait positive and negative affect and cardioception-related accuracy and confidence In fact, Bayesian analysis supported the lack of association for the majority of the analyses. The same was true for the two proprioceptive modalities. There are several possible explanations for these null-findings. Cardioceptive accuracy showed associations with arousal but not with valence in studies where emotions were experimentally evoked and the two dimensions were assessed independently (Wiens et al., 2000; Barrett et al., 2004; Pollatos et al., 2005, 2007b; Herbert et al., 2007, 2010; Köteles et al., 2020b). The approach used in the present study, however, primarily measures affective states that are accompanied with high arousal (Lox et al., 2010) thus cannot separate these components. Further, the actual affective state of participants was measured, which is necessary less intense than experimentally evoked affective states. Under such conditions, the already weak association between cardioception and emotional experience may disappear. Concerning chronic (trait-like) emotional states, previous studies assessed anxiety, an affective state accompanied with marked vegetative changes (i.e., sympathetic activation), particularly for patients with related disorders (e.g., Domschke et al., 2010). For positive and negative affect in healthy participants, however, the intensity of the emotions, including both the experience and the vegetative changes, are much lower. Also, we did not asses other interoceptive channels that might be associated with the emotional experience. This holds particularly true for proprioceptive accuracy, where the investigation of a single joint and task represent only one aspect of the proprioceptive accuracy of the whole body (Han et al., 2013). Secondly, emotions assessed with self-report might not be at the same level of consciousness as accuracy and confidence related decisions (Smith and Lane, 2015).

### Proprioception and Body-Related Questionnaires

Finally (H4), we replicated the findings on the independence of proprioceptive accuracy and interoceptive sensibility and body competence (Horváth et al., 2019) and extended them to another proprioceptive modality (weight discrimination) and confidence rating. The lack of association between interoceptive sensibility, a construct that integrates interoceptive experience across multiple channels, and interoceptive confidence ratings is particularly intriguing.

Also, our results indicate that the self-reported acute dimension of interoception is also modality-specific, i.e., cannot be generalized. As both constructs represent perceived abilities and the former is embedded in the latter (at least theoretically), their independence is clearly worthy of further investigation.

## Limitations

We investigated a sample of young people without known pathology; in this population, strong emotions were rarely presented. This leads to the decrease of variance in affective ratings which in turn makes the detection of associations difficult. The Schandry task has received considerable criticism recently (Ring et al., 2015; Desmedt et al., 2018, 2020; Ring and Brener, 2018; Zamariola et al., 2018); thus, although we applied a strict instruction which decreases the role of top-down factors, cardioception-related findings of the study might be flawed. Also, the joint reproduction task involves memory processes. Thus, cognitive abilities unrelated to interoception might also influence participants’ performance.

## Conclusion

Our findings indicate that interoceptive accuracy and confidence ratings are independent from each other in two proprioceptive modalities (joint reproduction with respect to the elbow joint and weight discrimination using the arm flexor muscles) and they are only weakly associated in the cardioceptive modality. There are no associations between accuracy and confidence ratings within the three interoceptive modalities. Finally, proprioceptive and cardioceptive accuracy and confidence ratings are not related to the acute and chronic affective state, interoceptive sensibility/body awareness and perceived body competence.

## Data Availability Statement

The raw data supporting the conclusions of this article will be made available by the authors, without undue reservation.

## Ethics Statement

The studies involving human participants were reviewed and approved by Research Ethics Committee of the Faculty of Education and Psychology at ELTE Eötvös Loraìnd University. The patients/participants provided their written informed consent to participate in this study.

## Author Contributions

All authors contributed to the conception and design of the study, read and commented on the last version of the manuscript. ÁH and LV contributed to the assessment of data. FK processed the data and performed the statistical analyses. ÁH wrote the first draft of the manuscript. EF and FK wrote sections of the manuscript. All authors contributed to the article and approved the submitted version.

## Conflict of Interest

The authors declare that the research was conducted in the absence of any commercial or financial relationships that could be construed as a potential conflict of interest.
